# Mycoplasma Pneumoniae Infection and Persistent Wheezing in Young Children: A Retrospective Case-Control Study

**DOI:** 10.3389/fped.2022.811086

**Published:** 2022-03-07

**Authors:** Huiming Sun, Shuxiang Li, Ting Wang, Zhengrong Chen

**Affiliations:** ^1^Department of Respiratory Medicine, Children's Hospital of Soochow University, Suzhou, China; ^2^Department of Nuclear Medicine, Suzhou Hospital Affiliated to Nanjing Medical University, Suzhou, China

**Keywords:** bronchoalveolar lavage, persistent wheezing, mycoplasma pneumoniae, children, serology, polymerase chain reaction

## Abstract

**Background:**

To investigate the clinical characteristics of children with persistent wheezing (PW) with Mycoplasma pneumoniae (MP) DNA in bronchoalveolar lavage fluid (BALF).

**Methods:**

This retrospective case-control study included consecutive admitted children under 3 years of age who were diagnosed with PW and had MP DNA detected in BALF. Patients with mycoplasma pneumoniae pneumonia (MPP) and foreign-body aspiration (FBA) were enrolled as controls. The clinical characteristics of the groups were compared.

**Results:**

During the study period, there were 89 patients diagnosed with PW without structural anomalies of the conductive airways, and 30 of these patients (33.7%, 30/89) with MP DNA detected in the BALF were selected as the study group. We included 44 patients with MPP and 44 patients with FBA as controls. Patients with MPP were older and had a higher occurrence of fever and C-reactive protein (CRP) than patients with PW (all *P* < 0.001). The median MP DNA copy number in patients with MPP was higher than that of patients with PW (*P* = 0.004). The median level of MP IgG in patients with PW was lower than that of patients with MPP and higher than that of patients with FBA (all *P* < 0.001). MP DNA copy number positively correlated with age (*r* = 0.392, *P* = 0.001) and CRP (*r* = 0.235, *P* = 0.048).

**Conclusions:**

Our study reveals that MP was highly detected in the BALF of PW patients. In addition, young patients with a low load of MP infection showed lower amounts of antibody, and a weak inflammatory response might be associated with PW.

## Introduction

It is believed that Mycoplasma pneumoniae (MP) causes a wide variety of respiratory diseases, including upper respiratory tract illnesses, bronchitis, atypical pneumonia and extrapulmonary diseases (1, 2). It is also believed that MP can trigger wheezing episodes, especially in young children (3, 4). Persistent wheezing (PW) is common in childhood and accounts for an extensive use of medical resources (5). However, most of the literature on PW has been case series studies, and comprehensive rigorous clinical research on PW is limited, resulting in poor knowledge of the physiopathology of PW (6). Studies have shown that MP can grow intracellularly and can establish chronic infection (1, 7). Previous studies suggested that chronic MP infection is related to chronic inflammation and persistent bronchial hyper-responsiveness in children and adults with asthma (8–10). Clinical evidence has demonstrated that MP is a pathogen of PW (11, 12). Treatment of patients with PW with conventional asthma therapy has not been completely effective (12), indicating that PW and asthma might have different physiopathologies.

In this study, we examined the clinical characteristics of children with PW with MP DNA detected in the bronchoalveolar lavage fluid (BALF) compared with those of patients with mycoplasma pneumoniae pneumonia (MPP) and foreign-body aspiration (FBA). We further explored the physiopathology of patients with PW infected by MP.

## Methods

This was a retrospective case-control study that included consecutive pediatric patients under 3 years of age who were diagnosed with PW and had MP DNA detected in BALF at the Children's Hospital of Soochow University from January 2013 to December 2019. The exclusion criteria were as follows: (1) gastroesophageal reflux; (2) congenital heart disease; (3) neuromuscular disorder; (4) born prematurely or birthweight <2,500 g; (5) airway structural abnormalities; (6) endobronchial tuberculosis; (7) family history of smoking; (8) immune deficiency; and (9) antibiotics use within 1 month before hospitalization.

We also enrolled consecutively admitted pediatric patients under 3 years of age diagnosed with MPP or FBA as control groups. The exclusion criteria were as follows: (1) congenital or acquired immunodeficiencies; (2) history of wheezing; (3) airway structural abnormalities; and (4) family or personal history of atopy. We chose patients with FBA as control group because performing flexible fiberoptic bronchoscopy in healthy patients is unethical. Patients with FBA were also excluded from the study if they had endoscopic bronchial inflammation.

This study was approved by the Ethics Committee of the Soochow University.

### Medical Data Collection

The following data were collected after admission: (1) demographic data, such as age and sex; (2) clinical data, such as fever and duration of hospitalization; (3) laboratory data, such as complete blood count, neutrophil ratio, lymphocyte ratio, platelets, C-reactive protein (CRP), and specific IgE detected from peripheral blood samples and obtained within 6 h after admission; (4) Indeed, because MP is frequently recovered in upper respiratory tract of normal children and serologic tests (IgG and IgM) are frequently positive for patients without any symptoms, the diagnosis of MP diseases is challenging (13), combined serology and PCR is the optimal test to confirm MP infection (14). Therefore, in our study, MP was detected using both BALF and serum samples; (5) adenovirus, respiratory syncytial virus, parainfluenza virus 1-3, influenza virus A and influenza virus B were detected with nasopharyngeal swab samples using direct fluorescent antibody test; rhinovirus, human metapneumovirus and bocavirus were also examined with nasopharyngeal swab samples by PCR; and bacteria were detected using BALF samples, with bacterial growth >10^4^ cfu/ml considered significant (11); and (6) For patients with MP infection, azithromycin is the preferred treatment (15), because it can be administered only once a day. In addition, this treatment requires fewer days and is associated with high compliance and tolerance. For patients with serious illness who need hospitalization, azithromycin (10 mg/kg/day) was prescribed intravenously for 5–7 consecutive days.

### Flexible Fiberoptic Bronchoscopy

A flexible fiber optic bronchoscope [Fujinon EB-270P (3.6 mm), Miyoshi, Japan or Olympus CV260 (2.8, 4.0 mm), Tokyo, Japan] was used according to China Respiratory Society recommendations, as previously described (16). The collected BALF was used for cell count and microbiological (MP and bacteria) analysis.

### Cell Counts

Cell counts were obtained using a modified version of Wright–Giemsa staining (Wright-Giemsa Stain, Baso Diagnostics Inc., China). At least 500 cells were examined. Data are expressed as a ratio of cells in total cell counts (16).

### Quantitative Detection of MP

A quantitative real-time PCR kit (DaAn Gene Co. Ltd., Guangzhou, China) was used to measure MP load; the assay was performed in accordance with the manufacturer's instructions (14). PCR amplification was performed using a 7600 RT PCR system (Applied Biosystems, Foster City, CA, USA). The detection limit was >2,500 copies/mL.

### MP Serology

MP specific antibodies (IgM and IgG) were detected using a commercial test kit (Shenzhen YHLO Biotech, Shenzhen, China). IgG > 24 RU/mL was considered positive, and IgM > 1.1 S/CO was considered positive (14).

### Clinical Definitions

PW was defined as wheezing lasting at least 4 weeks with no response to inhaled steroid and bronchodilator therapy (11). For patients with persistently focal crackles or with an axillary temperature >39.0°C, chest X-ray was needed to rule out pneumonia.

MPP was diagnosed when patients presented the following symptoms: (a) fever, irritant dry cough and dyspnea, wheezes or crackles, and pulmonary infiltrates on radiographs; and (b) MP DNA detected in BALF samples together with a positive IgM.

Fever was defined as an axillary temperature ≥38.0°C.

Allergic status was considered when an elevated specific IgE was found in blood.

### Statistical Analysis

Data analyses were performed using SPSS 21.0 software (IBM SPSS). We examined data normality using the Shapiro–Wilk test. Non-normal distribution data are presented as the medians [25–75th interquartile range (IQR)]. Statistical differences between groups were determined using non-parametric analyses (Mann–Whitney U test or the Kruskal–Wallis test). Differences in categorical variables were determined using the Chi-squared test. Correlations between the number of copies of MP DNA and the continuous variables were determined by Spearman correlation. *P*-values < 0.05 were considered statistically significant.

## Results

Between January 2013 and December 2019, there were 89 patients diagnosed with PW without structural anomalies of the conductive airways, and 30 of these patients (33.7%, 30/89) with MP DNA detected in the BALF were considered as the study group. In addition, 44 patients with MPP and 44 patients with FBA were considered as controls. In total, we enrolled 118 patients in the study. The patient age range was 3 to 35 months [median 18.0 (IQR (13.0–23.0)) months]. In the PW group, the budesonide dosages ranged from 1.0–2.0 mg daily, and the treatment duration [median (IQR)] was 1.0 (1.0–2.0) months.

The demographic and clinical characteristics of the enrolled patients are presented in Table 1. Compared with patients with PW, patients with MPP were older (*P* < 0.001) and had a higher occurrence of fever (*P* = 0.004), a higher neutrophil ratio (*P* = 0.003), a lower lymphocyte ratio (*P* = 0.007) and a higher CRP (*P* < 0.001). Compared with patients with PW, patients with FBA were older (*P* = 0.002) and had a lower occurrence of fever (*P* = 0.001). Compared with the patients with MPP, patients with FBA were younger (*P* < 0.001) and had a lower occurrence of fever (*P* < 0.001), a lower neutrophil ratio (*P* < 0.001), a higher lymphocyte ratio (*P* < 0.001) and a lower CRP (*P* < 0.001).

**Table 1:** Comparison of the clinical characteristics among patients with persistent wheezing, mycoplasma pneumoniae pneumonia and foreign-body aspiration.

**Parameters**	**Persistent wheezing group** **(*n* = 30)**	**Mycoplasma pneumoniae pneumonia group** **(*n* = 44)**	**Foreign-body aspiration group** **(*n* = 44)**	***P-*value**
Age, median (IQR), months	11.50 (7.75–19.0)	24.50 (16.50–29.75)^a^	17.0 (16.0–20.0)^b^^c^	<0.001
Male, *n* (%)	21 (70.0)	26 (59.1)	31 (70.5)	0.463
Fever, *n* (%)	18 (60.0)	39 (88.6)^a^	10 (22.7)^b^^c^	<0.001
**Whole blood cell analysis**				
Peripheral leukocyte count, median (IQR), × 10^9^/L	11.48 (7.81–14.61)	9.57 (7.28–12.55)	10.24 (8.86–12.53)	0.280
Neutrophil ratio, median (IQR), %	36.70 (29.48–47.75)	50.10 (36.65–66.28)^a^	35.60 (28.30–46.60)^c^	0.001
Lymphocyte ratio, median (IQR), %	52.85 (39.83–62.70)	37.90 (26.63–51.68)^a^	53.10 (42.70–62.50)^c^	0.001
Platelet number, median (IQR), 10^9^/L	408.0 (289.50–480.0)	317.50 (250.50–437.75)	313.0 (277.0–383.0)	0.089
C-reactive protein, median (IQR), mg/dL	1.22 (0.25–5.13)	9.95 (4.46–26.21)^a^	1.13 (0.32–4.08)^c^	<0.001

*IQR, interquartile range*.

a *Mycoplasma pneumoniae pneumonia group vs. persistent wheezing group, P < 0.05*.

b *Foreign-body aspiration group vs. persistent wheezing group, P < 0.05*.

c *Mycoplasma pneumoniae pneumonia group vs. foreign-body aspiration group, P < 0.05*.

**Figure 1:**
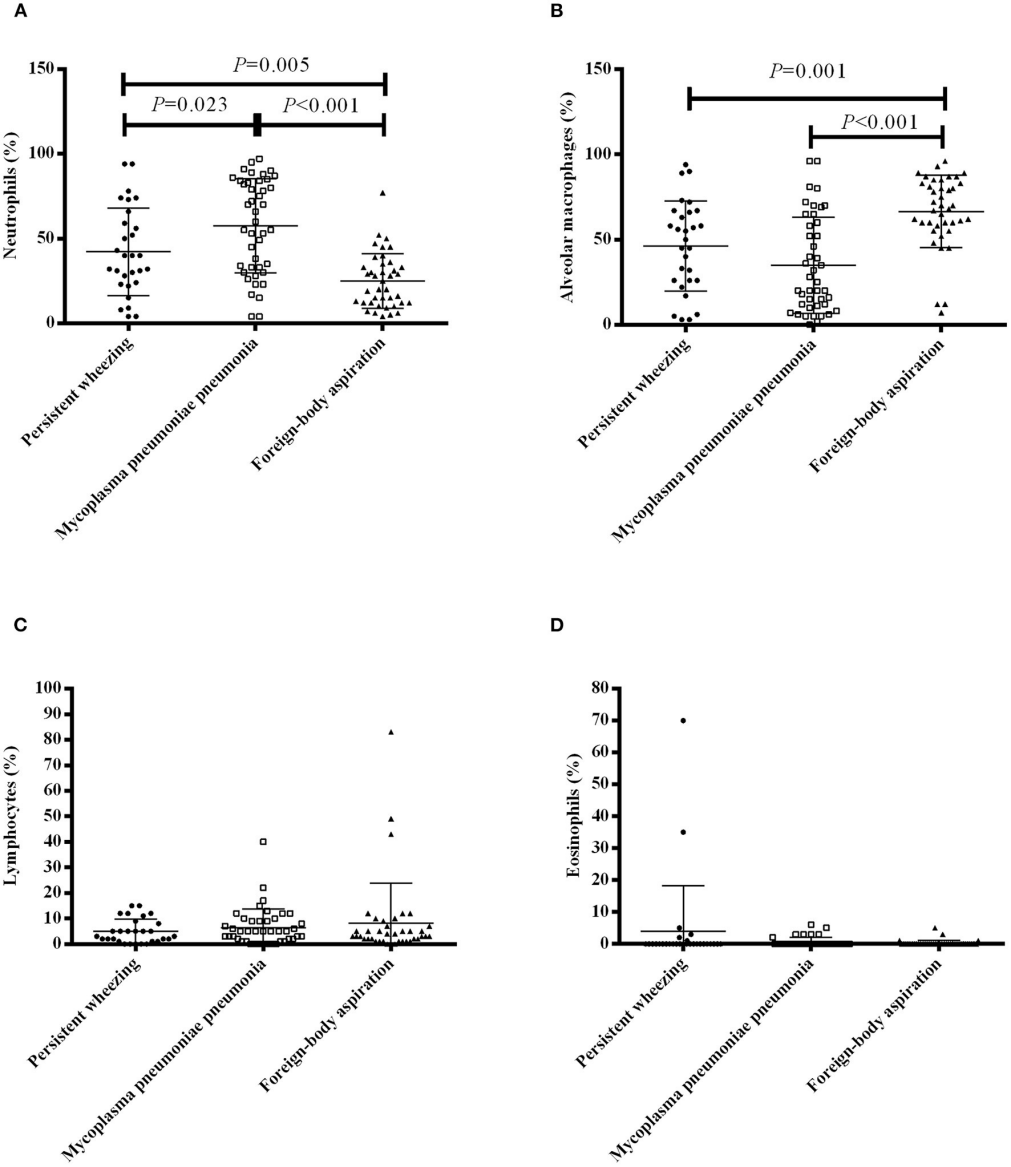
BALF cell profile in patients with PW, MPP and FBA. The percentages of neutrophils **(A)**, alveolar macrophages **(B)**, lymphocytes **(C)** and eosinophils **(D)** in bronchoalveolar lavage of patients with PW, MPP and FBA. Each dot, box and triangle indicates an individual patient.

The BALF cellular contents of the patients are presented in Figure 1. The bronchoalveolar lavage cell level in FBA patients was relatively normal. Patients with MPP had significantly higher neutrophil percentages compared with patients with PW [median 60.0% (IQR 33.0–84.0%) vs. 40.0% (23.5–62.5%), *P* = 0.023] and patients with FBA [median 60.0% (IQR 33.0–84.0%) vs. 22.5% (12.0–34.5%), *P* < 0.001] (Figure 1A). Patients with FBA had a significantly higher alveolar macrophage percentage compared with patients with PW [median 69.0% (IQR 58.5–83.0%) vs. 50.0% (26.0–66.5%), *P* = 0.001] and patients with MPP [median 69.0% (IQR 58.5–83.0%) vs. 25.0% (11.0–60.0%), *P* < 0.001] (Figure 1B). There were no significant differences in lymphocyte percentages (Figure 1C) and eosinophil percentages (Figure 1D) among patients with PW, patients with MPP and patients with FBA.

**Figure 2:**
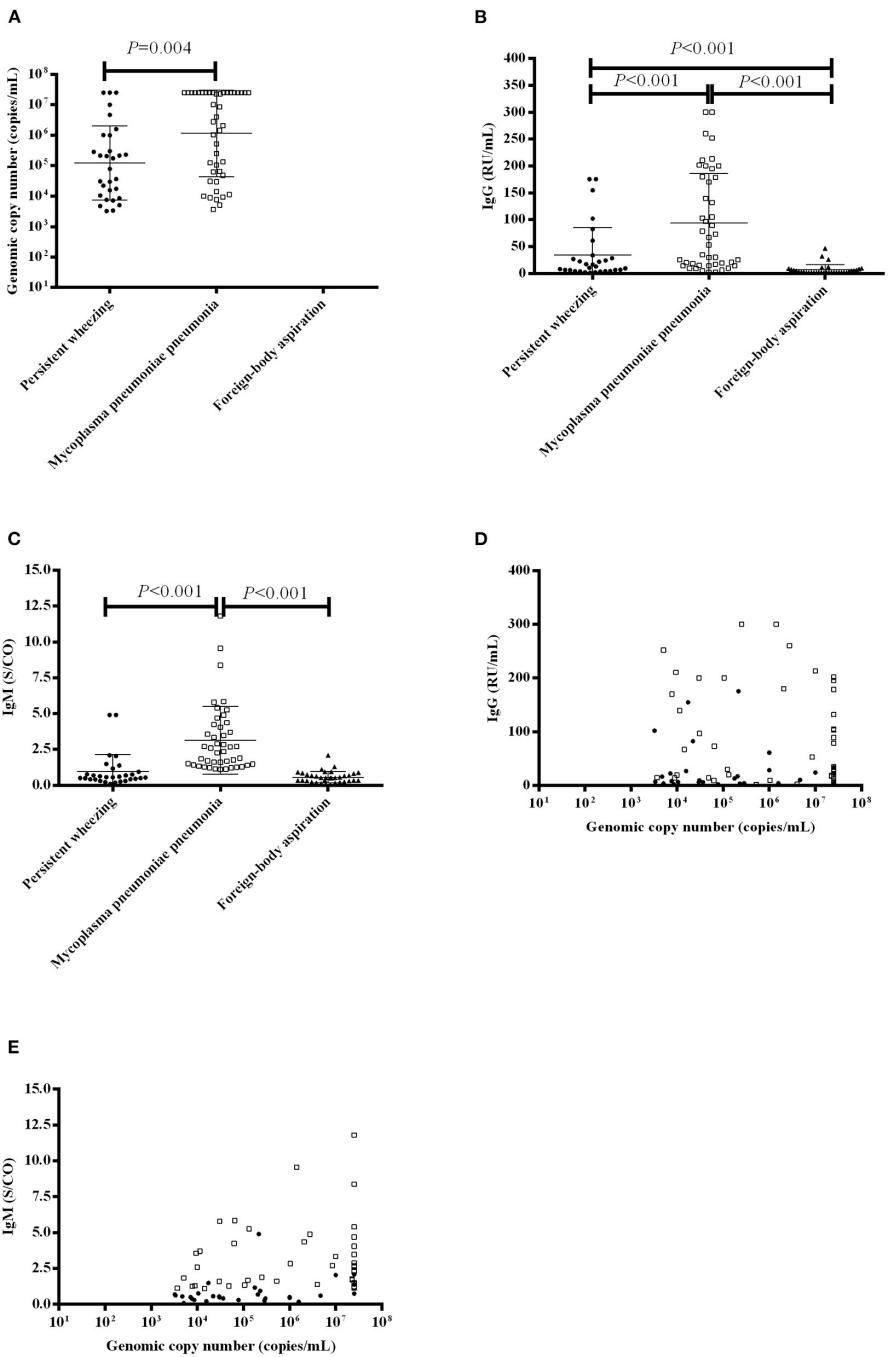
MP DNA loads and MP antibody levels in patients with PW, MPP and FBA. **(A)** MP DNA loads in the BALF of patients with PW, MPP and FBA; **(B)** Serum IgG levels in patients with PW, MPP and FBA; **(C)** Serum IgM levels in patients with PW, MPP and FBA; **(D)** Correlation of MP DNA loads and serum IgG levels; and **(E)** Correlation of MP DNA loads and serum IgM levels. Each dot, box and triangle indicates an individual patient.

MP DNA loads and MP antibodies levels are presented in Figure 2. The median BALF MP DNA copy number was higher in patients with MPP than that in patients with PW [median 3,380,000 copies/mL (IQR 51,900–25,000,000 copies/mL) vs. 126,550 copies/mL (10007.5–997,500 copies/mL), *P* = 0.004] (Figure 2A). The median level of MP IgG was higher in patients with MPP than patients with PW [median 60.17 RU/mL (IQR 15.65–179.78 RU/mL) vs. 11.86 RU/mL (4.58–29.81 RU/mL), *P* < 0.001] and patients with FBA [median 60.17 RU/mL (IQR 15.65–179.78 RU/mL) vs. 2.53 RU/mL (2.0–7.34 RU/mL), *P* < 0.001] (Figure 2B). The level of MP IgG was higher in patients with PW than patients with FBA [median 11.86 RU/mL (IQR 4.58–29.81 RU/mL) vs. 2.53 RU/mL (2.0–7.34 RU/mL), *P* < 0.001] (Figure 2B). The median level of MP IgM was higher in patients with MPP than patients with PW [median 2.47 S/CO (IQR 1.43–4.19 S/CO) vs. 0.56 S/CO (0.41–1.0 S/CO), *P* < 0.001] and patients with FBA [median 2.47 S/CO (IQR 1.43–4.19 S/CO) vs. 0.52 S/CO (0.31–0.83 S/CO), *P* < 0.001] (Figure 2C). The median level of MP IgM in patients with PW was not different from patients with FBA [median 0.56 S/CO (IQR 0.41–1.0 S/CO) vs. 0.52 S/CO (0.31–0.83 S/CO), *P* = 0.204] (Figure 2C).

We further evaluated the correlation between the MP DNA copy number and MP antibodies. We found that MP DNA copy number [median 267,000 copies/mL (IQR 209000–25000000 copies/mL)] was not correlated with IgG [median 13.10 RU/mL (IQR 3.94–64.21 RU/mL)] (*r* = 0.101, *P* = 0.393) (Figure 2D), but it positively correlated with IgM [median 0.96 S/CO (IQR 0.50–2.09 S/CO)] (*r* = 0.399, *P* < 0.001) (Figure 2E).

**Figure 3:**
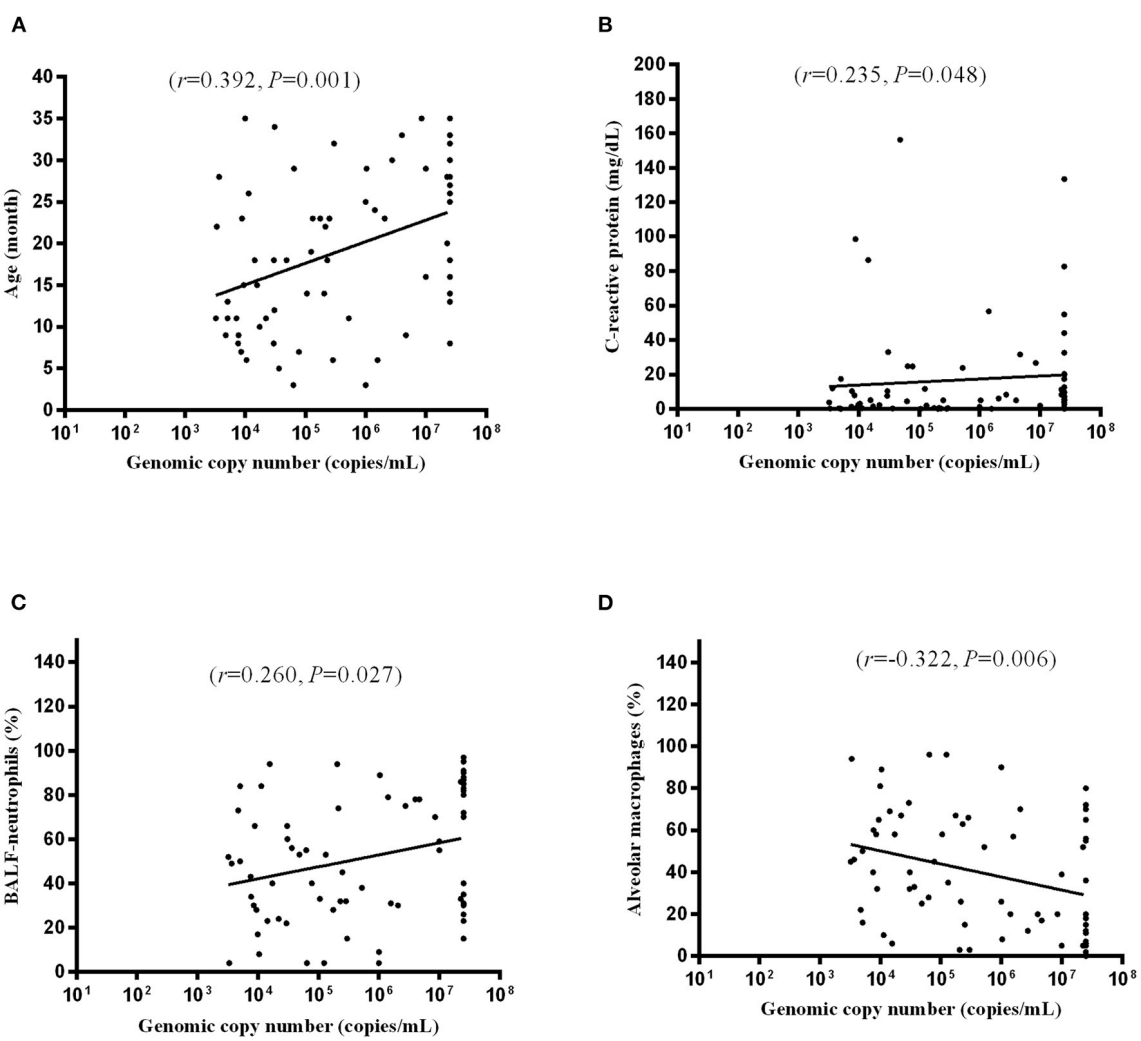
Correlation of MP DNA copy number and age **(A)**, CRP **(B)**, BALF-neutrophils **(C)**, and alveolar macrophages **(D)**. Each dot indicates an individual patient.

MP DNA copy number [median 267,000 copies/mL (IQR 209,000–25,000,000 copies/mL)] was positively correlated with age [median 18.0 months (IQR 13.0–23.0 months)] (*r* = 0.392, *P* = 0.001) (Figure 3A), CRP [median 3.08 mg/dL (IQR 0.46–11.85 mg/dL)] (*r* = 0.235, *P* = 0.048) (Figure 3B), and BALF–neutrophils [median 33.5% (IQR 20.0–66.0%)] (*r* = 0.260, *P* = 0.027) (Figure 3C) and inversely correlated with alveolar macrophages [median 55.5% (IQR 20.0–71.5%)] (*r*= −0.322, *P* = 0.006) (Figure 3D).

Among 30 patients with PW, specific IgE was detected in 16 patients: 7 patients had an elevated blood level of specific IgE and were considered allergic and 9 patients were non-allergic. Comparison of the BALF cellular contents between these two groups revealed no significant difference in the percentages of neutrophil, lymphocytes, eosinophils and alveolar macrophages in BALF between allergic and non-allergic patients (Table 2).

**Table 2:** BALF cellular content in allergic and non-allergic patients.

**Parameters**	**Allergic patients** **(*n* = 7)**	**Non-allergic patients** **(*n* = 9)**	***P-*value**
Neutrophil, %	40.0 (31.0–78.0)	30.0 (15.5–57.0)	>0.999
Lymphocytes, %	5.0 (2.0–8.0)	5.0 (0.0–12.0)	0.830
Eosinophils, %	0.0 (0.0–1.0)	0.0 (0.0–0.0)	0.485
Alveolar macrophages, %	56.0 (17.0–66.0)	57.0 (26.0–70.0)	0.312

*Data are presented as median (IQR)*.

The etiologic agents of the 30 patients with PW are presented in Table 3. MP alone was found in 14 patients, and mixed infection was found in 16 patients.

**Table 3:** Etiologic agents of 30 patients with persistent wheezing.

**Etiologic agent**	**Proportion (%) or *n***
Mycoplasma pneumoniae alone	14/30 (46.7)
Mixed infection	16/30 (53.3)
**Virus**	
Respiratory syncytial virus	2
Human bocavirus	4
Parainfluenzavirus type 3	1
Rhinovirus	2
Human metapneumovirus	1
Adenovirus	1
Respiratory syncytial virus + Rhinovirus	1
**Bacteria**	
*Escherichia coli*	1
*Staphylococcus aureus*	1
*Streptococcus pneumoniae* + *Moraxella catarrhalis*	1
**Virus + Bacteria**	
Human bocavirus + *Haemophilus influenzae*	1

All PW and MPP patients received intravenous azithromycin (10 mg/kg/day) for 5 consecutive days. The duration of hospitalization was not different between PW and MPP groups [median 10.0 days (IQR 8.0–13.0 days) vs. 9.0 days (8.0–12.75 days), *P* = 0.783]. Data of a 1-year follow-up study on these PW patients were available in 22 patients. Among these 22 patients, 9 (40.9%, 9/22) patients with PW experienced at least one episode of wheezing, and 13 (59.1%, 13/22) patients did not report wheezing episodes.

## Discussion

PW is common in childhood, and bronchial alveolar lavage is often suggested for the evaluation of children with PW. Notably, our study demonstrated that MP was highly detected in the BALF of PW patients. Therefore, clinicians should be alert to MP infection when treating patients with PW.

In our study, patients with MPP were older and had a higher occurrence of fever, a higher neutrophil ratio, a lower lymphocyte ratio and a higher CRP than patients with PW. These results indicate that patients with MPP have a strong inflammatory response compared with patients with PW, and these findings are consistent with those of previous studies (17, 18).

In our study, patients with MPP had a significantly higher BALF-neutrophil percentage than patients with PW. In addition, BALF MP DNA copy number was positively correlated with BALF-neutrophils. The interleukin-8 chemokine is involved in the recruitment of neutrophils (19). Lipid-associated membrane proteins from MP can stimulate pulmonary epithelial cells to express interleukin-8 *in vitro* (20). Increased interleukin-8 in the BALF was found in patients with MP lung infection (21), and the percentages of BALF neutrophils positively correlated with the levels of interleukin-8 in PW and asthma patients (22). Therefore, high MP loads in the BALF might induce high BALF-neutrophil percentages mediated by interleukin-8.

In our study, the median level of MP IgG in patients with PW was lower than that of patients with MPP but higher than that of patients with FBA. The median level of MP IgM in patients with PW was lower than that of patients with MPP, and the median BALF MP DNA copy number was lower in patients with PW than that of patients with MPP. MP possesses both glycolipid and protein antigens that elicit antibody responses, and MP-specific antibodies contribute to eliminate MP after infection (1). Previous studies reported that chronic MP infection may occur among patients with humoral immunodeficiency after initial infection (23–25). Our findings may suggest that lower loads of MP infection elicit lower antibody responses, leading to the chronic carriage of MP.

Our results showed that MP DNA copy number was positively correlated with age and CRP. These findings indicate that young patients had a lower load of MP infection compared with older patients, which might lead to a lower inflammatory response.

We did not detect any significant difference in the percentages of neutrophil, lymphocytes, eosinophils and alveolar macrophages in the BALF between allergic and non-allergic PW patients. This result suggests that the BALF cell profile in PW patients might not correlate with atopic status, which is in agreement with the results of a previous study (26).

In our study, the duration of hospitalization was not different between PW and MPP groups. One reason might be that MP RNA clearance was reported as faster among patients with PW after azithromycin treatment (27).

Our study had several limitations. First, we did not set a control group of patients with acute MP bronchitis. However, performing flexible fiberoptic bronchoscopy in patients with MP bronchitis is unethical. A previous study reported the mean level of CRP in infants with acute MP bronchitis was 8.5 mg/dL (28). In our study, the median level of CRP in patients with PW was 1.22 mg/dL, indicating that patients with acute MP bronchitis might have a stronger inflammatory response than patients with PW. Second, we did not explore the mechanisms underlying the neutrophil induction by MP in the BALF of patients with MPP and PW. Third, we used relatively stringent exclusion criteria and therefore small numbers of subjects were included in this study. Therefore, clinical studies with larger samples are needed in the future. Fourth, in our study, viruses were detected in 12 (40.0%, 12/30) patients with PW. Because these viruses were detected with nasopharyngeal swab samples, it was difficult to distinguish what was the real cause of PW. Therefore, further studies are needed to detect these viruses in the BALF. Fifth, although our study argued for a role for MP in both situations, there was no evidence that antibiotic treatment had a positive impact on outcome. Finally, the percentage of eosinophils in the BALF was not different between allergic and non-allergic PW patients. However, we did not detect activated and degranulated eosinophils, and further studies are needed to examine eosinophil cationic protein levels in the BALF between the two groups.

In conclusion, our findings revealed that MP was highly detected in the BALF of PW patients. Young patients with a low load of MP infection may elicit lower amounts of antibodies, and a weak inflammatory response might be associated with PW.

## Data Availability Statement

The raw data supporting the conclusions of this article will be made available by the authors, without undue reservation.

## Ethics Statement

This study was approved by the Ethics Committee of the Soochow University.

## Author Contributions

HS and SL summarized the data and wrote the article. TW collected the patient's information. ZC designed the study, analyzed data, and revised the manuscript. All authors read and approved the final manuscript.

## Funding

This work was supported by the National Natural Science Foundation of China (Grant Number: 81900006), the Social Development Projects of Jiangsu Province (Grant Number: BE2019671), and the Livelihood science and technology of Suzhou (Grant Number: SS201869).

## Conflict of Interest

The authors declare that the research was conducted in the absence of any commercial or financial relationships that could be construed as a potential conflict of interest.

## Publisher's Note

All claims expressed in this article are solely those of the authors and do not necessarily represent those of their affiliated organizations, or those of the publisher, the editors and the reviewers. Any product that may be evaluated in this article, or claim that may be made by its manufacturer, is not guaranteed or endorsed by the publisher.

## References

[1] Waites KB Talkington DF. Mycoplasma pneumoniae and its role as a human pathogen. Clin Microbiol Rev. (2004) 17:697–728, table of contents. 10.1128/CMR.17.4.697-728.2004 15489344 PMC523564

[2] Narita M. Pathogenesis of extrapulmonary manifestations of Mycoplasma pneumoniae infection with special reference to pneumonia. J Infect Chemother. (2010) 16:162–9. 10.1007/s10156-010-0044-X 20186455

[3] Rhim JW Kang HM Yang EA Lee KY. Epidemiological relationship between Mycoplasma pneumoniae pneumonia and recurrent wheezing episode in children: an observational study at a single hospital in Korea. BMJ Open. (2019) 9:e026461. 10.1136/bmjopen-2018-026461 30975681 PMC6500193

[4] Biscardi S Lorrot M Marc E Moulin F Boutonnat-Faucher B Heilbronner C. Mycoplasma pneumoniae and asthma in children. Clin Infect Dis. (2004) 38:1341–6. 10.1086/392498 15156467

[5] Martinez FD Wright AL Taussig LM Holberg CJ Halonen M Morgan WJ. Asthma and wheezing in the first six years of life. the group health medical associates. N Engl J Med. (1995) 332:133–8. 10.1056/NEJM199501193320301 7800004

[6] Ren CL Esther CR Debley JS Sockrider M Yilmaz O Amin N. Official American thoracic society clinical practice guidelines: diagnostic evaluation of infants with recurrent or persistent wheezing. Am J Respir Crit Care Med. (2016) 194:356–73. 10.1164/rccm.201604-0694ST 27479061

[7] Dallo SF Baseman JB. Intracellular DNA replication and long-term survival of pathogenic mycoplasmas. Microb Pathog. (2000) 29:301–9. 10.1006/mpat.2000.0395 11031124

[8] Wood PR Hill VL Burks ML Peters JI Singh H Kannan TR. Mycoplasma pneumoniae in children with acute and refractory asthma. Ann Allergy Asthma Immunol. (2013) 110:328–34. 10.1016/j.anai.2013.01.022 23622002 PMC3703852

[9] Sutherland ER King TS Icitovic N Ameredes BT Bleecker E Boushey HA. A trial of clarithromycin for the treatment of suboptimally controlled asthma. J Allergy Clin Immunol. (2010) 126:747–53. 10.1016/j.jaci.2010.07.024 20920764 PMC2950827

[10] Varshney AK Chaudhry R Saharan S Kabra SK Dhawan B Dar L. Association of mycoplasma pneumoniae and asthma among Indian children. FEMS Immunol Med Microbiol. (2009) 56:25–31. 10.1111/j.1574-695X.2009.00543.x 19239491 PMC7110376

[11] Gu W Jiang W Zhang X Chen Z Yan Y Huang L. Refractory wheezing in Chinese children under 3 years of age: bronchial inflammation and airway malformation. BMC Pediatr. (2016) 16:145. 10.1186/s12887-016-0680-0 27568177 PMC5002096

[12] De Schutter I Dreesman A Soetens O De Waele M Crokaert F Verhaegen J. In young children, persistent wheezing is associated with bronchial bacterial infection: a retrospective analysis. BMC Pediatr. (2012) 12:83. 10.1186/1471-2431-12-83 22726254 PMC3420249

[13] Spuesens EB Fraaij PL Visser EG Hoogenboezem T Hop WC van Adrichem LN. Carriage of Mycoplasma pneumoniae in the upper respiratory tract of symptomatic and asymptomatic children: an observational study. PLoS Med. (2013) 10:e1001444. 10.1371/journal.pmed.1001444 23690754 PMC3653782

[14] Li QL Dong HT Sun HM Zhang XX Gu WJ Huang L. The diagnostic value of serological tests and real-time polymerase chain reaction in children with acute mycoplasma pneumoniae infection. Ann Transl Med. (2020) 8:386. 10.21037/atm.2020.03.121 32355830 PMC7186703

[15] Subspecialty Subspecialty Group of Respiratory Diseases TSoPCMA Editorial Board CJoP. [Guidelines for management of community acquired pneumonia in children (the revised edition of 2013) (I)]. Zhonghua er ke za zhi. (2013) 51:745–52. 10.3760/cma.j.issn.0578-1310.2013.10.006 24406226

[16] Sun H Li S Yan Y Chen Z Wang Y Hao C. Associations between patient clinical characteristics and the presence of cytomegalovirus DNA in the bronchoalveolar lavage fluid of children with recurrent wheezing. BMC Infect Dis. (2018) 18:458. 10.1186/s12879-018-3345-9 30200894 PMC6131915

[17] Sun H Chen Z Yan Y Huang L Wang M Ji W. Epidemiology and clinical profiles of Mycoplasma pneumoniae infection in hospitalized infants younger than one year. Respir Med. (2015) 109:751–7. 10.1016/j.rmed.2015.04.006 25957828

[18] Defilippi A Silvestri M Tacchella A Giacchino R Melioli G Di Marco E. Epidemiology and clinical features of mycoplasma pneumoniae infection in children. Respir Med. (2008) 102:1762–8. 10.1016/j.rmed.2008.06.022 18703327

[19] Altstaedt J Kirchner H Rink L. Cytokine production of neutrophils is limited to interleukin-8. Immunology. (1996) 89:563–8. 10.1046/j.1365-2567.1996.d01-784.x 9014822 PMC1456568

[20] Choi SY Lim JW Shimizu T Kuwano K Kim JM Kim H. Reactive oxygen species mediate Jak2/Stat3 activation and IL-8 expression in pulmonary epithelial cells stimulated with lipid-associated membrane proteins from Mycoplasma pneumoniae. Inflamm Res. (2012) 61:493–501. 10.1007/s00011-012-0437-7 22270622

[21] Bohnet S Kotschau U Braun J Dalhoff K. Role of interleukin-8 in community-acquired pneumonia: relation to microbial load and pulmonary function. Infection. (1997) 25:95–100. 10.1007/BF02113584 9108184 PMC7101691

[22] Hauk PJ Krawiec M Murphy J Boguniewicz J Schiltz A Goleva E. Neutrophilic airway inflammation and association with bacterial lipopolysaccharide in children with asthma and wheezing. Pediatr Pulmonol. (2008) 43:916–23. 10.1002/ppul.20880 18668688

[23] Block S Hedrick J Hammerschlag MR Cassell GH Craft JC. Mycoplasma pneumoniae and Chlamydia pneumoniae in pediatric community-acquired pneumonia: comparative efficacy and safety of clarithromycin vs. erythromycin ethylsuccinate. Pediatr Infect Dis J. (1995) 14:471–7. 10.1097/00006454-199506000-00002 7667050

[24] Taylor-Robinson D Webster AD Furr PM Asherson GL. Prolonged persistence of Mycoplasma pneumoniae in a patient with hypogammaglobulinaemia. J Infect. (1980) 2:171–5. 10.1016/S0163-4453(80)91284-0 6821085

[25] Dumke R Jacobs E. Antibody response to mycoplasma pneumoniae: protection of host and influence on outbreaks? Front Microbiol. (2016) 7:39. 10.3389/fmicb.2016.00039 26858711 PMC4726802

[26] Le Bourgeois M Goncalves M Le Clainche L Benoist MR Fournet JC Scheinmann P. Bronchoalveolar cells in children <3 years old with severe recurrent wheezing. Chest. (2002) 122:791–7. 10.1378/chest.122.3.791 12226015

[27] Chen J Ji F Yin Y Yuan S. Time to mycoplasma pneumoniae RNA clearance for wheezy vs. non-wheezy young children with community-acquired pneumonia. J Trop Pediatr. (2021) 67:fmaa109. 10.1093/tropej/fmaa109 33274390

[28] Wang Y Hao C Ji W Yan Y Shao X Xu J. Bronchiolitis associated with mycoplasma pneumoniae in infants in Suzhou China between 2010 and 2012. Sci Rep. (2015) 5:7846. 10.1038/srep07846 25597274 PMC4297954

